# A genome-wide association study of copy-number variation identifies putative loci associated with osteoarthritis in Koreans

**DOI:** 10.1186/s12891-015-0531-4

**Published:** 2015-04-04

**Authors:** Sanghoon Moon, Bhumsuk Keam, Mi Yeong Hwang, Young Lee, Suyeon Park, Ji Hee Oh, Yeon-Jung Kim, Heun-Sik Lee, Nam Hee Kim, Young Jin Kim, Dong-Hyun Kim, Bok-Ghee Han, Bong-Jo Kim, Juyoung Lee

**Affiliations:** Division of Structural and Functional Genomics, Center for Genome Science, National Institute of Health, 363-951 Chungchengbuk-Do, Republic of Korea; Department of Internal Medicine, Seoul National University Hospital, 110-744 Seoul, Republic of Korea; Department of Social and Preventive Medicine, Hallym University College of Medicine, 200-702 Chunchun, Republic of Korea; Department of Biostatistics, Soonchunhyang University, College of Medicine, 140-743 Seoul, Republic of Korea

**Keywords:** Osteoarthritis, Copy number variation, OA risk gene, TNKS, CA10

## Abstract

**Background:**

OA is a complex disease caused by environmental and genetic risk factors. The purpose of this study is to identify candidate copy number variations (CNVs) associated with OA.

**Methods:**

We performed a genome-wide association study of CNV to identify potential loci that confer susceptibility to or protection from OA. CNV genotyping was conducted using NimbleGen HD2 3 × 720K comparative hybridization array and included samples from 371 OA patients and 467 healthy controls. The putative CNV regions identified were confirmed with a TaqMan assay.

**Results:**

We identified six genomic regions associated with OA encompassing CNV loci. None of six loci had previously been reported in genome-wide association studies with OA, although a genetic analysis suggested that they have functional effects. The protein product of a candidate risk gene for obesity, *TNKS*, targets Wnt inhibition, and this gene was significantly associated with hand and knee OA. Copy number deletion on *TNKS* was associated with a 1.37-fold decreased risk for OA. In addition, *CA10*, which shows a strong association with osteoporosis, was also significant in our study. Copy number deletion on this gene was associated with a 1.69-fold decreased risk for OA.

**Conclusion:**

We identified several CNV loci that may contribute to OA susceptibility in Koreans. Further functional investigations of these genes are warranted to fully characterize their putative association.

**Electronic supplementary material:**

The online version of this article (doi:10.1186/s12891-015-0531-4) contains supplementary material, which is available to authorized users.

## Background

Osteoarthritis (OA), which is also known as degenerative arthritis, is a disease of mechanical dysfunction involving degradation of joint components, especially articular cartilage and subchondral bone [1]. OA is the most common form of arthritis that affects elderly people and often leads to physical disability and reduced quality of life. OA is a leading cause of impaired mobility in the elderly [2]. The clinical course of OA varies from minimal joint pain, stiffness, and limitations in function to complete disability.

Development of OA is the consequence of interaction between external stimuli, such as mechanical loading and the structure and physiology of the joint [3]. Risk factors of OA include obesity, knee injury, previous knee surgery, and occupational bending and lifting [4]. Symptoms may include joint pain, tenderness, stiffness, locking, and sometimes an effusion from the affected joint. Various causes including genetic predisposition, developmental, metabolic, and mechanical deficits can initiate inflammatory processes that lead to loss of cartilage and clinically overt OA. Not only mechanical and inflammatory stimuli to joint but also genetic susceptibility to such stimuli contribute to the pathogenesis of OA.

Recently, a number of genetic association studies for OA have been performed using candidate gene approaches or genome-wide association studies (GWASs). Several genes have been identified as associated with OA. *GDF5* [5,6], *DUS4L* [7], *7q22* [8], *MCF2L* [9] and *GNL3* [10] meet the strict criteria of genome-wide significance level with a P-value of < 5 ×10^-8^. In addition to these genes, several candidate single-nucleotide polymorphisms (SNPs) in *FRZB, DIO2,* and *SMAD2* are regarded as potentially associated genes [11-13]. However, the considerable number of SNPs in OA most likely reflects the polygenic nature of the disease with modest effect sizes for individual genes [14] and may be related to the somewhat inconclusive findings of these studies. Hence, novel approaches beyond GWASs are warrant.

Copy number variations (CNVs) account for a major proportion of human genetic polymorphisms and have been predicted to play an important role in genetic susceptibility to common diseases [15]. CNVs are one type of promising structural variation beyond SNPs, which affect only a single nucleotide. CNVs may range from ~1 kilobase to several megabases in size and can result in cellular dysfunction. CNVs associated with OA have not been elucidated.

The purpose of this study is to identify candidate CNVs associated with OA. To examine the contribution of a CNV region to OA, we carried out a genome-wide association analysis between selected CNV regions and OA. This study revealed potential genetic variants that had not been previously reported from GWASs.

## Methods

### Study population

A total of 10038 individuals from the Korean Genome and Epidemiology Study, which is an ongoing prospective community-based epidemiology study in the communities of Ansung (rural) and Ansan (urban) were included. From that group, 838 individuals (371 OA cases and 467 healthy controls) who consented to X-ray examination were genotyped with NimbleGen HD2 3 × 720K comparative genomic hybridization array (aCGH). All the participants including controls provided written informed consent. And the study was approved by the ethics committee of Korea Centers for Disease Control and Prevention Institutional Review Board. The basic characteristics of the study participants are summarized in Table 1.

**Table 1:** **Basic characteristics of study subjects**

**OA phenotype**	**Number**		**Gender (%women)**			**Age (mean)**			**BMI** ^**a**^ **(mean)**		
	**Cases**	**Controls**	**Cases**	**Controls**	***P*** **-value**	**Cases**	**Controls**	***P*** **-value**	**Cases**	**Controls**	***P*** **-value**
ROA One definite osteophyte (original K ≥2)	371	467	84%	80%	0.1974	62.22	61.23	0.023	25.55	24.28	1.3 × 10^-7^

BMI^a^: body mass index.

### Diagnostic criteria

OA phenotype was diagnosed based on clinical and radiographic findings without assessment of symptoms. Alternative methods of assessment such as self-reported and symptomatic OA could not be used in this study. Participants who agreed to undergo X-rays had both wrists and both knees examined. Radiographic OA was assessed based on the Kellgren/Lawrence (K/L) grading system. Patients with K/L grades of 2 or higher in the wrists or knees were diagnosed as OA [16].

### CNV genotyping platform

NimbleGen 3 × 720 aCGH was used for CNV calling. This platform provides more than 720000 probes. A total of 360000 probes distributed uniformly across the entire human genome, whereas another 360000 probes distributed on targeted location. The median inter-probe spacing of the backbone is < 5 kb. DNA from the NA10851 cell line was used as a reference for the aCGH to extract signal intensity ratio with hg18/NCBI build 36. To adjust systemic biases that are present in microarray experiment, all of samples passed experimental control metrics such as chromosome X shift and mad.1dr with NimbleScan v.2.5. After quality control, signal intensity ratio of each probe was converted into log2 scale with the positions of probes.

### CNV calling

To conduct reliable CNV association analyses, we divided the CNV calling process into two stages: CNV detection and CNV genotyping (Additional file 1). For CNV detection, the Genome Alteration Detection Analysis algorithm (GADA) was used for CNV discovery with T = 10, alpha = 0.2, and MinSegLen = 10 [17]. An average log_2_ ratio of ±0.25 of probes was set as a cut-off value to define the CNV region. To overcome the limitation of single algorithm detection, we tested CNV discovery with several parameters to find best parameter using known CNV region. Consequently, we select best parameter with highly concordant with known CNV region.

### Genotype estimation

We used the software package, CNVtools with default parameters to assign individuals to each CNV genotype [18]. Particularly, estimated genotype from a linear discrimination function (LDF) of CNVtools was used for association analysis (Additional file 2). A mixture-based model used in CNVtools can separate samples into more exact CNV genotype group. To do this, all individuals of each CNV regions from the CNV detection with GADA were clustered according to the log_2_ ratio between test sample and reference sample. Additional file 3 shows type of CNV class. It is not easy to discriminant exact CNV genotype in the single-class CNVs, in which all individuals of the CNV region belonged to one cluster. On the contrary, multi-class CNVs that consisted of more than two clusters can assign highly confident CNV genotype. Therefore, only well-clustered multi-class CNV regions were included for further study. All CNV regions were also manually inspected.

Given low frequency or rare CNVs, if the number of individuals in a cluster was more than three (tripleton), we regarded this cluster as an independent cluster, whereas if the number of individuals in a cluster was two or fewer (doubleton), we did not consider this cluster as a new cluster.

### Statistical analysis

Statistical analyses were performed to identify significant variant associations with hand and/or knee OA using the R package version 3.0.2. Logistic regression analysis adjusting for gender, age and BMI as covariates was used to calculate the statistical significance for cases-control analysis. Regarding the genotype coding scheme, 0 and 1 were used for two-class CNV regions. Heterozygous deletion (one copy) and normal (two copies) were coded as 0 and 1, respectively. For three-class CNV regions, homozygous deletion (zero copies), heterozygous deletion (one copy) and normal (two copies) were coded as 0, 1, and 2, respectively.

### Validation of CNVs

To verify estimated CNV regions, we carried out quantitative PCR using the TaqMan Copy Number Assay (Life Technologies, Foster City, USA) according to the manufacturer’s protocols. In total, four pre-designed and two customized probes were used to validate the existence of the CNV. Validation samples (cases and controls) were randomly selected from each estimated genotype cluster. All validation experiments were replicated 3 times to increase the validation accuracy. The copy number in each individual was calculated with Copy Caller v2.0 using the comparative CT method according to the manufacturer’s protocols.

## Results

### Study population of each OA type

A total of 371 patients (310 women, 61 men) were thus diagnosed as having OA (Table 1). The proportion of female was 84% in cases and 80% in controls, respectively. Among 371 cases, 204 and 167 were cases of hand and knee OA, respectively (Table 2).

**Table 2:** **Number of cases and controls in each OA type**

**Study**	**Classification system**	**Cut-off value for OA**	**Exact OA definition**	**Knee OA**		**Wrist OA**	
				**Cases**	**Controls**	**Cases**	**Controls**
KARE^a^	K/L score	2	One definite osteophyte (original K ≥2)	167	467	204	467

^a^KARE: the Korea Association Resource project.

### Selection of candidate CNV regions

To estimate the reliable CNV genotype of each individual, we selected multi-class CNV regions that were well separated between clusters. Additional file 3 shows an example of a multi-class cluster that we selected. In total, 1123 multi-class CNV regions were selected for the association study. Among these, 463 CNV regions were two-class regions (one and two copies) and 660 were three-class regions (zero, one and two copies) (Additional file 3). In addition, we mainly focused on copy-number deletion rather than copy-number duplication due to convenience of detection and validation.

### Statistical analysis

We conducted logistic regression analyses between identified CNV regions and OA. Consequently, we selected six candidate CNV loci that were associated with OA and that ranged from about ~1 kb to 18.5 kb (Table 3). Four CNV regions were within genic region (intron), and the other two regions were in non-genic region (inter-genic). After adjusting for gender, age and BMI, the P-values of the six CNV regions still satisfied a nominal P-value threshold of < 0.05 in hand and/or knee OA.

**Table 3:** **Summary of association result**

**Chr**	**Start**	**End**	**Length (bp)**	**Gene**	**OA type**	**CNV genotype frequency (%)** ^**a**^		**OR (95% CI)** ^**b**^	**Adjusted P-value** ^**c**^
						**Cases (0/1/2)**	**Controls (0/1/2)**		
1	112490851	112509390	18539	Upstream of *KCND3*	Hand	23.0/52.9/24.0	13.1/52.5/34.5	0.41 (0.25 - 0.68)	5.8 × 10^-4^
8	9535412	9536779	1367	Intron of *TNKS*	Hand, Knee	2.4/28.8/68.7	4.3/34.5/61.2	1.37 (1.02 - 1.85)	3.5 × 10^-2^
9	71952096	71953157	1061	Intron of *MAMDC2*	Knee	-/7.2/92.8	-/3.0/97.0	0.36 (0.16 - 0.86)	1.9 × 10^-2^
11	85981796	85983048	1252	Intron of *ME3*	Hand	6.9/33.3/59.8	11.1/40.5/48.4	1.70 (1.21 - 2.40)	2.4 × 10^-3^
13	36971042	36976531	5489	Downstream of *POSTN*	Hand, Knee	2.4/31.3/66.3	1.5/22.5/76.0	0.64 (0.47- 0.87)	4.9 × 10^-3^
17	47477132	47478158	1026	Intron of *CA10*	Hand	1.5/19.1/79.4	2.8/26.6/70.7	1.69 (1.14 - 2.55)	1.02 × 10^-2^

^a^Designations for the CNV genotype are as indicated in the Methods. A dash indicates a zero-copy CNV region. OR^b^ and Adjusted P-value^c^ were adjusted by gender, age, and body mass index; OR: odds ratio, CI: Confidence interval.

### Experimental validation to assess accuracy

To assess the accuracy of CNV estimation, we performed validation experiments with the six significant CNV regions. Table 4 shows the validation results of these six regions. The positive predictive value was applied as a measurement standard of accuracy and indicated the proportions of positive results in the CNV validations. The average positive predictive value of our CNV validation results was 0.973, indicating that the CNV genotype estimation was highly reliable. Additional file 4 shows the TaqMan assay result of the CNV on chromosome 8. The estimated CNV genotype (Additional file 4 (A)) was highly concordant with the validated genotype (Additional file 4 (B)). Except for the CNV region on chromosome 9, the genotypes of the five regions were zero, one, and two copies (Table 4).

**Table 4:** **Result of TaqMan copy number assays**

**Chr**	**Start**	**End**	**Probe ID**	**CNV genotype** ^**a**^	**TP**	**FP**	**PPV** ^**b**^
Chr1	112490851	112509390	Hs_03201731_cn	0, 1, 2	179	9	0.95
Chr8	9535412	9536779	Hs_04345744_cn	0, 1, 2	73	1	0.99
Chr9	71952096	71953157	Hs_06899364_cn	1, 2	58	1	0.98
Chr11	85981796	85983048	Hs_03777027_cn	0, 1, 2	70	4	0.95
Chr13	36971042	36976531	CCT956B	0, 1, 2	85	3	0.97
Chr17	47477132	47478158	CCD1TXG	0, 1, 2	86	0	1.00

^a^Designations for the CNV genotype are as indicated in the Methods. ^b^PPV: positive predictive value, PPV is defined as number of TPs/(number of TPs + number of FPs).

## Discussion

In this study we conducted a genome-wide association study of CNVs in OA to identify genomic regions that confer susceptibility and/or protection. The genome-wide significance level following Bonferroni correction for the 1123 CNV regions in this study was 4.5 × 10^-5^. Because not every variant that has a functional impact always satisfies a genome-wide significant level and because relatively small sample size can undermine the reliability of true variants, some variants with evidence obtained from genetic study are suggested to have functional effects, even though none of identified variants meet a genome-wide significance level. Thus, we investigated functional relationships between OA and CNV regions that reached nominal significance (P < 0.05). Consequently, we identified six OA genomic regions that confer susceptibility and/or protection. These regions included *TNKS, CA10*, *POSTN, MAMDC2, KCND3,* and *ME3* which encompassed or were near CNV regions.

The canonical Wnt/β-catenin signaling pathway has been implicated in the pathogenesis of osteoarthritis [19]. Increased β-catenin accumulations have been observed in degenerative cartilage, suggesting that activated Wnt signaling might contribute to progression of osteoarthritis by downstream target genes such as matrix metalloproteinases (MMPs) [20]. For instance, the secreted Wnt antagonist Dickkopf-1 is related with slowed progression of hip OA in elderly women [21] and the small molecule XAV939, which selectively inhibits β-catenin-mediated transcription, is associated with protecting cartilage degradation in a rat osteoarthritis model [22]. Tankyrase, which is encoded by *TNKS*, bind directly to axin, a negative regulator of the canonical Wnt/β-catenin signaling pathway, forming a destruction complex with glycogen synthase kinase 3β (GSK-3β) and adenomatous polyposis coli (APC) to degrade β-catenin [23]. Inhibition of *TNKS* by treating the compound XAV939 or small interfering mediated silencing of *TNKS* can cause an increase in levels of axin, leading to phosphorylation and degradation of β-catenin, and inhibition of target gene transcription by Wnt signaling. Thus, although further studies are needed to establish the role of *TNKS* in osteoarthritis through axin-mediated inhibition of Wnt signaling, it is quite possible that *TNKS* could be risk factor for osteoarthritis.

**Figure 1:**
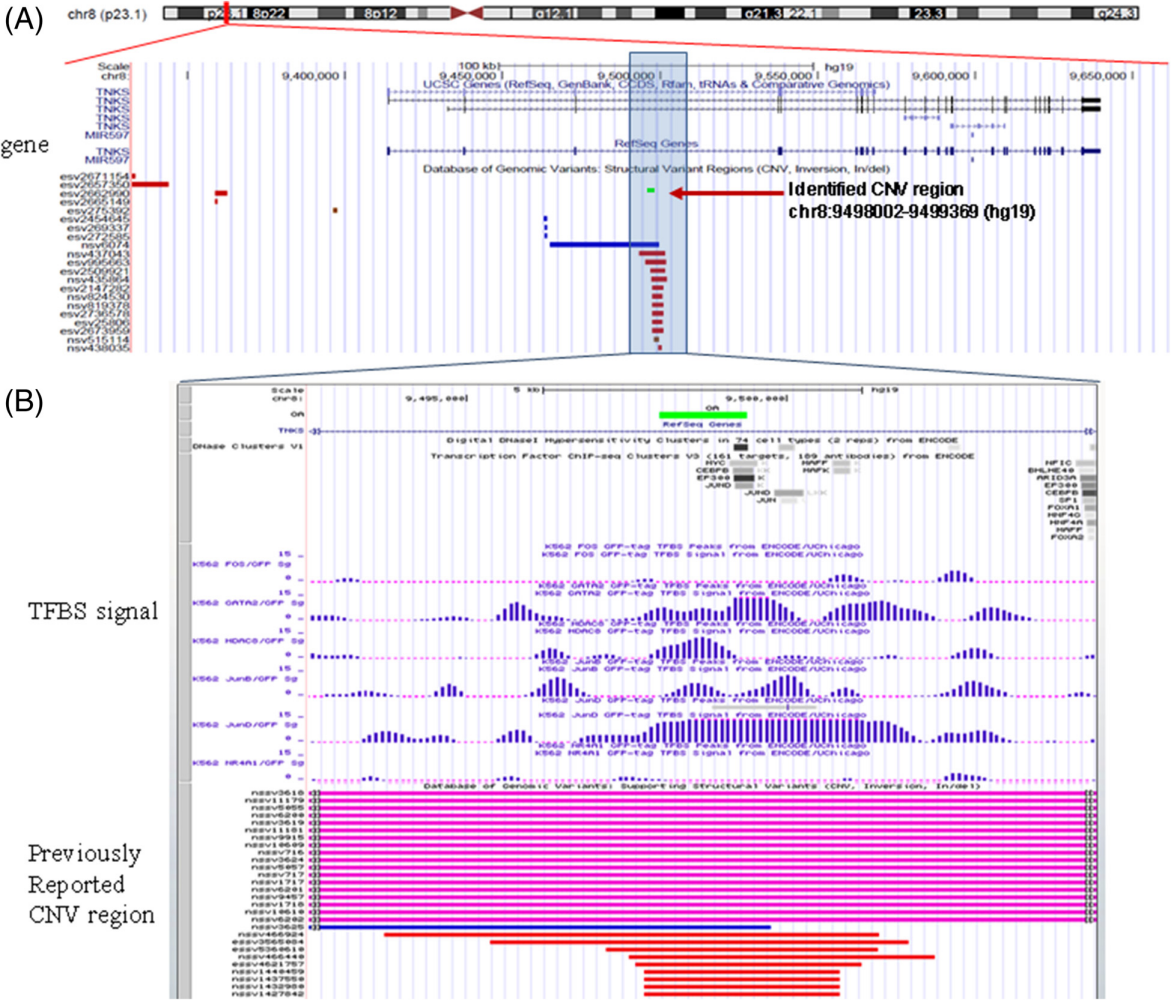
**A copy-number deletion at chromosomal position 8p23.1.** The detected CNV region in this study is marked by green filled square. The coordinate of the CNV region were recalculated based on hg19 by liftOver of UCSC genome browser. **(A)** CNV region overlapped with previously reported CNV regions from Database of Genomic Variants. **(B)** Several transcription factor binding sites as determined by ENCODE were found within this CNV region, indicating that copy-number deletion of this region may influence genetic regulation.

Figure 1 shows the chromosomal position of the copy number deletion of third intron of *TNKS*. Interestingly, several transcription factor binding sites as determined by ENCODE of UCSC genome browser (http://genome.ucsc.edu/ENCODE) overlapped with this CNV region (Figure 1B). We speculated that copy number deletion of this region may directly or indirectly influence the binding of transcription factors, possibly including JunD, which belongs to the Jun family of activator protein 1 transcriptional regulators. A recent study reported that JunD-deficient immortalized fibroblast exhibit increased proliferation [24]. JunD also regulates *interleukin-6* and *matrix metalloproteinase-9* transcription in hepatic stellate cells, indicating that JunD may be a key profibrogenic regulator [25]. Copy number deletion of TNKS was associated with a 1.37-fold decreased risk for OA (Table 2).

*CA10* gene encodes a protein that belongs to the carbonic anhydrase family of zinc metalloenzymes, which catalyze the reversible hydration of carbon dioxide [26]. The protein encoded by *CA10* is an acatalytic member of the alpha-carbonic anhydrase subgroup, which includes major players in many physiological processes including renal and male reproductive tract acidification and bone resorption. Carbonic anhydrases are thought to be involved in bone mineral solubilization and digestion of organic bone matrix by acid proteases. The rapid conversion of carbon dioxide to bicarbonate and protons occurs while maintaining a normal intracellular pH in osteoclasts [27]. The use of an inhibitor of carbonic anhydrase is associated with a bone-sparing effect, as shown by measurement of spinal bone mineral density [28]. Moreover, the intronic variants, rs2106329 in *CA10* shows strong association with osteoporosis in Japanese women [29]. A homozygous A allele at rs2106329 is a protective factor for osteoporosis. In our study, copy number deletion on *CA10* was associated with a 1.69-fold decrease risk for hand OA as compared with a normal copy number (Table 3). Copy number deletion can also be a protective factor for OA.

*POSTN* encodes periostin, an osteoblast-specific factor that functions as a ligand for αV/β3 and αV/β5 integrins to support adhesion and migration of epithelial cells [30]. Periostin is a matricellular glutamate-containing protein that is expressed in connective tissues including bone, periosteum, and ligament [31]. Periostin is believed to function in the regulation of bone formation and, with its preferential localization in the periosteum, may play a role in fracture repair during the early process of bone regeneration [31]. In response to mechanical stress, periostin expression is up-regulated and collagen fibrillogenesis and matrix organization occur to preserve tissue integrity and function [32]. Periostin knock-out mice show altered cortical bone microarchitecture and lower bone mineral density [33]. Periostin appears to influence the properties of bone materials and damage accumulation and repair, including local modeling/remodeling processes in response to fatigue [34]. Periostin deficiency may influence the propensity for fatigue fractures [34], and may accelerate the pathogenesis of OA in cooperation with an imbalance in bone repair mechanisms. Similar to previously reported study, in our study, copy number deletion downstream of *POSTN* was associated with an increase in the risk for hand and/or knee OA compared with the normal copy number. However, we did not find a relationship between this CNV region and *POSTN*.

Also, we could not find a biological role of *MAMDC2, KCND3,* and *ME3* in the pathogenesis of OA.

The present study has some limitations. First, diagnosis of OA was radiologically based (i.e., using X-ray) without assessment of symptoms. Different methods of assessment such as self-reported and symptomatic OA could not be available in population-based study samples. This limitation has resulted in small sample size of discovery stage and a difficulty of collecting samples for replication analysis. Second, because of first limitation which was mentioned above, we did not success to find radiographic case samples in independent general population samples. Eventually, we did not conduct a replication study, which is highly desirable with association studies. This limitation can be solved through a meta-analysis with other data in the future. To reduce heterogeneity of meta-analysis, we provide our cohort information such as exact phenotype definition, cut-off, and number of cases of each OA type. Third, knee and hand OA were analyzed together. OA of the knee can be a component of a generalized diathesis, including OA of the hand, which may be inherited. The OA phenotype is heterogeneous and occurs at specific joint locations, in a more generalized form, or with and without clinical presentation. Each phenotype has its own heritability estimate [35]. Fourth, the sample size was relatively small, and this reduced the power of the analysis. Fifth, six novel CNVs were located not in exons but in introns or were intergenic. Despite these limitations, our study has the following strengths. First, the present study describes the first CNVs to be reported regarding OA using high-resolution chip. Second, we were able to define precise disease phenotype using X-rays of all the participants.

## Conclusions

We identified several previously unreported CNV loci that predisposed to or provided protection from OA. Identification of the associated variants at these genomic loci and their potential functional consequences will lead to better understanding of the pathogenesis of OA. Although this study has some limitations, further functional investigations of these genes are warranted to fully characterize the association.

## Additional files

Additional file 1:
**Overall scheme of this study.** A genome-wide association study of CNVs was conducted using 371 of OA cases and 467 controls. Consequently, six candidate CNV regions were selected. Additional file 2:
**Result of CNVtools with estimated genotypes and Log2 ratio information.** We used LDF value among three different genotype information such as raw, PCA, and LDF. PCA: Principal components analysis, LDF: linear discrimination function. Additional file 3:
**CNV classes.** For the CNV genotyping stage, we assigned individuals to each CNV cluster according to the log_2_ ratio between test sample and reference sample. (A) Single-class CNVs, in which all individuals of the CNV region belonged to one cluster, were excluded from further study. (B, C) Only multi-class CNVs that consisted of two (B) or three (C) clusters were used for the association analysis. Additional file 4:
**Genotypes and validation results of the CNV region on** ***TNKS*** **. (A)** The Histogram represents the signal intensity of log_2_ ratio for OA cases (left) and controls (right). CNV genotypes of this region were clearly separated into three groups (homozygous deletion, heterozygous deletion and normal). The colored lines (black, green and cyan) show the posterior probability for each of the three copy number classes (homozygous deletion, heterozygous deletion and normal copy). **(B)** Quantitative PCR results showed that the validated genotype was highly concordant with estimated CNV genotype. The copy number state of cases (left) and control samples (right). Higher bar, lower bar and no bar in each figure represent a normal number of copies, heterozygous deletion, and homozygous deletion, respectively. The Blue bar means copy number state of the NA10851 sample, which was used as the reference sample.

## Abbreviations

OA: Osteoarthritis
GWAS: Genome-wide association study
SNP: Single nucleotide polymorphism
CNV: Copy number variation
aCGH: Comparative genomic hybridization array
BMI: Body mass index
