# Supplementary figures and images for: A genome-wide association study of copy-number variation identifies putative loci associated with osteoarthritis in Koreans
Source: BMC Musculoskelet Disord. 2015 Apr 4;16:76. doi: 10.1186/s12891-015-0531-4 (PMC4395893; doi:10.1186/s12891-015-0531-4)

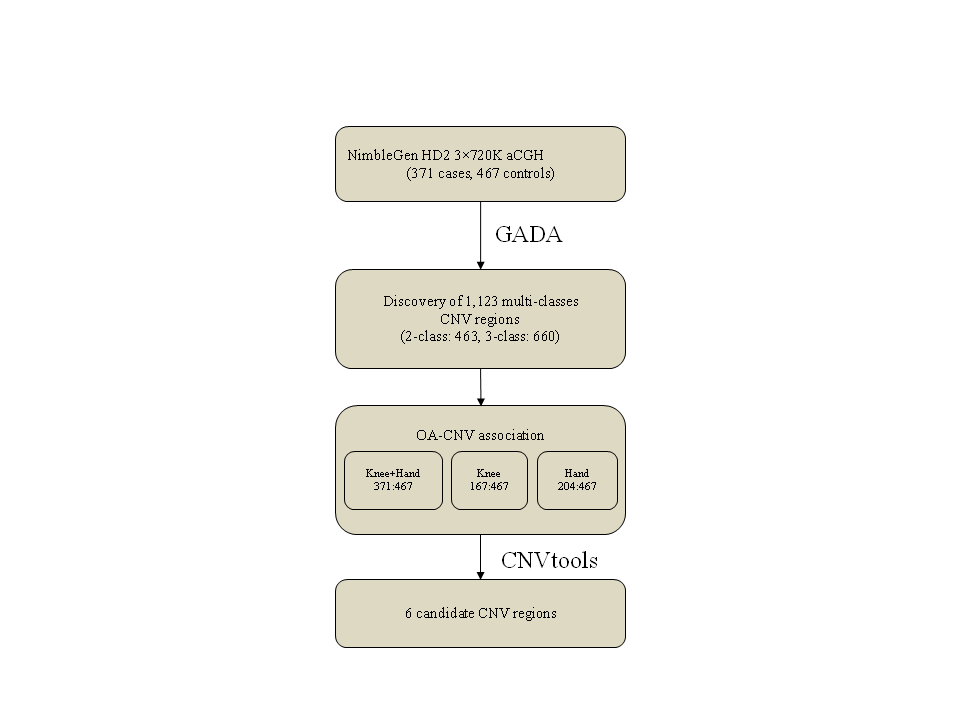

Supplement: Additional file 1: — Overall scheme of this study. A genome-wide association study of CNVs was conducted using 371 of OA cases and 467 controls. Consequently, six candidate CNV regions were selected. [file 12891_2015_531_MOESM1_ESM.tiff]

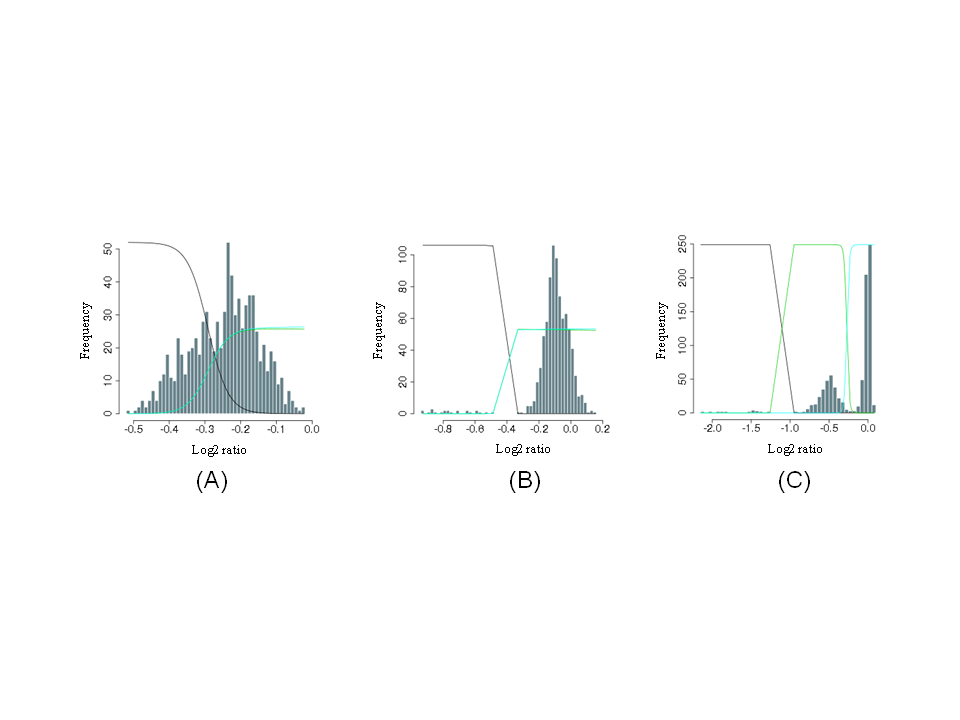

Supplement: Additional file 3: — CNV classes. For the CNV genotyping stage, we assigned individuals to each CNV cluster according to the log2 ratio between test sample and reference sample. (A) Single-class CNVs, in which all individuals of the CNV region belonged to one cluster, were excluded from further study. (B, C) Only multi-class CNVs that consisted of two (B) or three (C) clusters were used for the association analysis. [file 12891_2015_531_MOESM3_ESM.tiff]

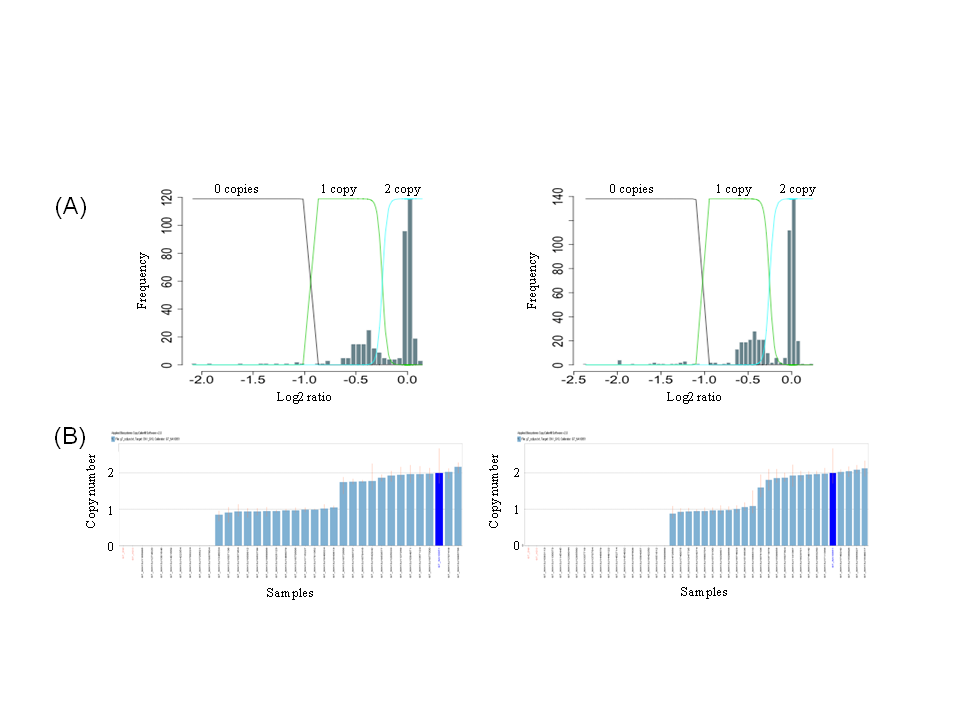

Supplement: Additional file 4: — Genotypes and validation results of the CNV region on TNKS . (A) The Histogram represents the signal intensity of log2 ratio for OA cases (left) and controls (right). CNV genotypes of this region were clearly separated into three groups (homozygous deletion, heterozygous deletion and normal). The colored lines (black, green and cyan) show the posterior probability for each of the three copy number classes (homozygous deletion, heterozygous deletion and normal copy). (B) Quantitative PCR results showed that the validated genotype was highly concordant with estimated CNV genotype. The copy number state of cases (left) and control samples (right). Higher bar, lower bar and no bar in each figure represent a normal number of copies, heterozygous deletion, and homozygous deletion, respectively. The Blue bar means copy number state of the NA10851 sample, which was used as the reference sample. [file 12891_2015_531_MOESM4_ESM.tiff]
